# Metagenomic and biogenic amine changes in cassava fermentation for tucupi production using *Pediococcus acidilactici* starter culture

**DOI:** 10.1002/jsfa.70350

**Published:** 2025-11-25

**Authors:** Danielle Gama Brício, Maria Beatriz A Gloria, José Augusto Pires Bitencourt, Leandro Araujo Argôlo, Rafaela de Lima Ribeiro, Ilzane Abreu da Silva, Rosinelson da Silva Pena, Alessandra Santos Lopes, Gilson CA Chagas Junior, Nelson Rosa Ferreira

**Affiliations:** ^1^ Programa de Pós‐graduação em Ciência e Tecnologia de Alimentos Instituto de Tecnologia, Universidade Federal do Pará Belém Brazil; ^2^ Laboratório de Controle de Qualidade, Faculdade de Farmácia Universidade Federal de Minas Gerais Belo Horizonte Brazil; ^3^ Instituto Tecnológico Vale Belém Brazil; ^4^ Projeto Bionorte, Programa de Pós‐graduação em Biodiversidade e Biotecnologia Belém Brazil; ^5^ Present address: Programa de Pós‐Graduação em Ciência de Alimentos, Faculdade de Farmácia, Universidade Federal da Bahia Ondina, Salvador, Bahia 40.170‐115 Brazil

**Keywords:** *Weissella*, *Lactococcus*, putrescine, histamine, PCA, HCA

## Abstract

**BACKGROUND:**

The use of starter cultures is essential for producing fermented foods with desirable standardized characteristics and for preventing pathogens. *Pediococcus acidilactici*, isolated from cocoa fermentation, was used in the production of tucupi, a widely appreciated sauce made from the juice of cassava root (manipueira) in the Brazilian Amazon. Manipueira was submitted to fermentation with and without *Pediococcus acidilactici* inoculum at 1 × 10^12^ CFU mL^−1^ (Pa treatment and control treatment (CT), respectively), over a 24‐h period. Samples were collected at 4‐h intervals and analyzed for physicochemical characteristics following official methods, bioactive amines by high‐performance liquid chromatography with fluorescence detection (HPLC‐FLD), and microbial genera identification by metagenomic analysis.

**RESULTS:**

Physicochemical results indicated that fermentation took its due course, with increased acidity, as well as lower pH and reducing and total sugars (Tukey test, *P* ≤ 0.05). Only two biogenic amines were detected (putrescine and histamine), and higher levels were found in Pa treatment compared to CT, probably due to the increased *Lactobacillus* prevalence. Six genera were identified in CT (*Weissella*, *Lactobacillus*, *Lactococcus*, *Leuconostoc*, *Bacillus*, and *Enterococcus*), whereas seven were in Pa (*Weissella*, *Lactobacillus*, *Pediococcus*, *Lactococcus*, *Leuconostoc*, *Streptococcus*, and *Enterococcus*). *Weissella*, which was predominant in manipueira, decreased during fermentation, whereas *Lactobacillus* became predominant in CT. However, when *P. acidilactici* was used, *Lactobacillus* was prevalent throughout fermentation, and there was a reduced prevalence of *Bacillus* and *Enterococcus,* bacteria with pathogenic potential.

**CONCLUSION:**

The starter culture optimized tucupi production by maintaining low levels of biogenic amines, inhibiting the growth of undesirable microorganisms, and enhancing tucupi quality and safety. © 2025 The Author(s). *Journal of the Science of Food and Agriculture* published by John Wiley & Sons Ltd on behalf of Society of Chemical Industry.

## INTRODUCTION

Cassava (*Manihot esculenta* Crantz) is a perennial woody plant of the Euphorbiaceae family, known for its adaptability to diverse ecological and agronomic conditions, including poor soils and drought, unlike many other crops. It is widely cultivated and serves as a staple food for millions of people.^1^, ^2^ On average, cassava's composition consists of 93.9% moisture, 4.8% carbohydrates, 0.5% protein, 0.5% ash, and 0.3% lipids.^3^ Cassava has great nutritional and cultural importance in Brazil. It is very versatile, with extensive culinary use of fresh root and derived products, including flour, starch, tapioca, and tucupi.^4^

A yellowish liquid (manipueira) is separated from the solids during cassava root pressing for flour production. Manipueira undergoes natural fermentation followed by cooking, resulting in tucupi.^5^ Tucupi is a key ingredient in Pará's cuisine (Brazilian Amazon), widely used in traditional dishes such as *tacacá* and duck in tucupi.^6^ According to Normative Instruction (NI) number 001/2008, tucupi is defined as a product or by‐product derived from cassava roots through an appropriate technological process. This regulation also specifies its sensory characteristics and establishes physicochemical (e.g., pH values and sugar content) and microbiological standards, including hygienic‐sanitary requirements.^7^

Studies on tucupi began in the last decades, primarily focusing on its physicochemical characteristics^8^ and cyanide safety.^9^ However, limited information is available on key aspects such as processing, nutrient composition, and safety. Brito *et al*.^5^ initiated efforts to better characterize tucupi, reporting, for the first time, the presence of carotenoids (9‐*cis*‐*β*‐carotene, all‐*trans*‐*β*‐carotene, and 13‐*cis*‐*β*‐carotene) and bioactive amines (spermidine, putrescine, histamine, and tyramine). While carotenoids and some amines are inherent to the cassava species,^5^ other amines may be formed during processing, especially under inadequate hygienic‐sanitary conditions.^10^

Brito *et al*.^11^ found that commercially available tucupi contained high levels of undesirable amines (e.g., putrescine and histamine). The presence of high levels of these amines in tucupi is undesirable. Putrescine can impart a putrid smell to tucupi,^12^ whereas high histamine levels can cause adverse health effects, including histamine poisoning (no‐observed‐adverse‐effect level (NOAEL) of 50 mg per meal per individual), with symptoms including headaches, vomiting, dizziness, and anaphylaxis.^13^ However, histamine intolerance, a condition caused by the inability to digest histamine in the diet, is becoming prevalent, with allergy‐like symptoms. NOAEL recommended for histamine intolerance is no detectable limit levels.^13^, ^14^ Therefore, any amount of histamine in food could cause histamine intolerance.

The use of starter cultures in fermented products provides advantages from the safety and quality control points of view. It enhances safety by inhibiting the growth of undesirable spoilage and pathogenic microorganisms. In addition, it improves quality through the consistent production of desirable flavors and aromas. It extends product shelf life. In addition, it enables predictable fermentation, ensuring consistent product quality and reducing processing time by competing with the indigenous microorganisms and accelerating desirable biochemical changes.^15^ The production of tucupi is still an artisanal process, following spontaneous fermentation. There is scarce scientific information regarding the use of starter cultures during tucupi processing. However, the occurrence of biogenic amines in tucupi has been reported.^5^, ^11^ Therefore, by using a starter culture for tucupi production, it is likely that a more standardized tucupi is produced with lower levels of biogenic amines.

The use of a starter culture during tucupi processing could benefit from all the earlier‐mentioned advantages, as well as the improvement of safety regarding histamine and other biogenic amines. Briefly, it outcompetes endogenous amine‐producing bacteria for nutrients and creates an acidic environment that inhibits their growth.^16^

Therefore, a potential approach to improve tucupi quality is to inhibit the growth of pathogenic, spoilage, and biogenic amines‐forming microorganisms, assuring safety and preventing the formation and accumulation of undesirable biogenic amines.^17^ This could be achieved using a starter culture, such as lactic acid bacteria (LAB), that rapidly produces organic acids, inhibiting undesirable microbial growth and extending shelf life.^18^ Additionally, some LAB can help prevent the formation of harmful biogenic amines.^19^, ^20^ *Pediococci* are LAB that have been widely used as starter cultures in the food industry. Some *Pediococcus* spp. strains can produce bacteriocins and other antimicrobial metabolites.^21^, ^22^ In addition, *Pediococcus acidilactici* has been reported to significantly reduce the occurrence of biogenic amines in fermented foods.^19^, ^23^ Recently, this LAB was isolated and identified during cocoa fermentation in the Brazilian Amazon,^24^ where its presence was associated with the inhibition of the formation of putrefactive amines, for example, putrescine and cadaverine. The availability of *P. acidilactici* from the native microbiota of a traditional fermented product, such as cocoa, that possesses desired metabolic and safety characteristics, can benefit tucupi quality, safety, and shelf life.^16^ Another advantage of using *P. acidilactici* is associated with its probiotic potential. *Pediococcus acidilactici* is an acid‐ and bile‐tolerant bacterium with intestinal adherence properties. In addition, it maintains a symbiotic relationship with other microorganisms, protecting the host against pathogens by stimulating protective immunoglobulins. It also regulates immune cells and reduces intestinal bacterial translocation.^25^

Studies on the microbiota of tucupi are scarce. Microbiological analysis has focused on compliance with hygienic‐sanitary conditions and safety requirements,^7^ showing adequate results. Recently, a study^26^ investigated the active microbial dynamics during tucupi fermentation by means of metaproteomic analysis. They observed that during fermentation, LAB was prevalent, including *Lactobacillus*, *Lactococcus*, and *Limosilactobacillus*. However, metagenomic analysis can examine all genetic material in the sample, regardless of cell viability, and it can also identify the function of each detected gene.^27^

As mentioned previously, tucupi is a food product containing high levels of biogenic amines. The formation of amines can result from the native microbiota, likely due to the lack of good manufacturing practices and deficient hygienic‐sanitary conditions, typical of artisanal processing. Studies on other fermented foods, such as soy products^25^ and fish fillets,^28^ have highlighted *P. acidilactici* as an effective tool to prevent or reduce the growth of undesirable biogenic amine‐producing microorganisms, including those generating putrescine, histamine, and cadaverine. Cavalcante *et al*.^26^ reported that the addition of *P. acidilactici* starter culture modulated the functional profile of fermentative microorganisms in cassava, favoring the glycolytic pathway for pyruvate production, which serves as the basis for energy generation and the synthesis of ethanol and other volatile compounds in tucupi, such as acetaldehyde.

In this context, the objective of this study was to provide molecular identification of the predominant microorganisms during manipueira fermentation and to investigate the influence of *P. acidilactici* as a starter culture during manipueira fermentation for tucupi production regarding the microbial dynamics and the formation and occurrence of biogenic amines. It is expected that the use of this starter culture will improve the microbiological profile, physicochemical properties, and biogenic amines levels of the product. Thus, the tucupi industry could benefit from this approach to produce a higher‐quality, safer, and standardized tucupi.

## MATERIAL AND METHODS

### Reagents, chemicals, and samples

Reagents and chemicals (analytical grade) were from Neon (Suzano, SP, Brazil) and Exôdo Científica (Sumaré, SP, Brazil). For high‐performance liquid chromatography (HPLC) analysis of bioactive amines, the standards (spermine tetrahydrochloride, spermidine trihydrochloride, putrescine dihydrochloride, agmatine sulfate, cadaverine dihydrochloride, 5‐hydroxytryptamine‐serotonin, histamine dihydrochloride, tyramine hydrochloride, 2‐phenylethylamine hydrochloride, tryptamine), sodium acetate, sodium octane sulfonate, *o*‐phthalaldehyde, and ultrapure water were purchased from Merck Group (Burlington, MA, USA). Acetonitrile for HPLC grade was from J.T. Baker (Phillipsburg, NJ, USA). For chromatographic analysis, HPLC solvents were filtered through a 0.45 μm nylon membrane (Millipore, Burlington, MA, USA). For microbiological analysis, peptone water, Man, Rogosa and Sharpe (MRS) broth, and MRS agar were obtained from Kasvi (São José dos Pinhais, PR, Brazil).

Manipueira (48 L) was obtained at the Ver‐o‐Peso market in the city of Belém (PA, Brazil) (coordinates 01°27′08″ S, 48°30′13″ W) and transported under refrigeration to the Laboratory of Biotechnology Processes (LAPROBIO) at the Federal University of Pará (UFPA).

### *Pediococcus acidilactici* strains and starter culture preparation

The starter culture was prepared with the LAB *P. acidilactici* (GenBank Access MT117910),^21^ from the Microorganism Bank of LAPROBIO/UFPA and registered in the Brazilian National System for the Management of Genetic Heritage and Associated Traditional Knowledge (SisGen, AF31270). The microorganism was stored under freezing (−18 °C) in MRS broth containing 15% glycerol.

The strain was reactivated by inoculation of a 10 μL aliquot in a test tube containing 10 mL of sterile MRS broth, followed by incubation for 24 h in a bacteriological incubator at 35 °C. The technique used to produce the starter culture was described by Chagas Junior *et al*.^17^ with a few modifications (anaerobic conditions). A 10 mL aliquot of active culture was inoculated into an Erlenmeyer flask containing 90 mL of sterile MRS broth. After this period, the inoculum was transferred to a benchtop bioreactor (FerMac 320; Electrolab Biotech, Tewkesbury, UK) with 900 mL of sterile MRS broth, and it was kept under agitation of 150 rpm, at 35 °C until cell counts reached 10^12^ cells mL ^−1^. The final contents were centrifuged at 10 000 × *g* for 14 min at 4 °C. The supernatant was discarded, and the pellet was kept refrigerated (4 °C) for 24 h before use. After weighing, the pellet was transferred to the manipueira fermentation vessels.

### Fermentation of manipueira and tucupi production

Sterilized polyethylene vessels were used for fermentation. Manipueira (8 L) was fermented without inoculum (control treatment, CT), and, in another vessel (8 L), manipueira was fermented with 1 × 10^12^ CFU mL^−1^ of *P. acidilactici* (Pa treatment). The containers were covered with aluminum foil and incubated at 30.0 ± 2.0 °C for 24 h. The experiment was performed in triplicate.

The process was monitored through physicochemical analysis, with samples collected at 4 h intervals (0, 8, 12, 16, 20, and 24 h). After 24 h of fermentation, the fermented manipueira was heat‐treated at ~95 °C for 40 min to obtain tucupi.^3^ Samples were taken throughout the fermentation process and of the final product (tucupi) for physicochemical, metagenomic, physicochemical, and bioactive amines analyses.

In tucupi‐producing regions, a 24‐h fermentation of manipueira is a long‐standing traditional practice, proven to be safe and effective for developing desirable sensory attributes and reducing hydrocyanic acid (HCN) levels.^3^ The inoculum level applied followed Chagas Junior *et al*.,^17^ who successfully used similar concentrations in other Amazonian fermentations, such as cocoa bean processing.

### Methods of analysis

#### Physicochemical analysis of manipueira and tucupi

Aliquots of manipueira samples from the fermentation vessels and the final tucupi from both CT and Pa treatments were analyzed according to the Association of Official Analytical Chemists.^29^ The samples were submitted to pH, total titratable acidity, reducing sugars, total sugars, and soluble solids analysis. All the analyses were performed in triplicate. These parameters were used to ensure that fermentation took place accordingly.

#### DNA extraction and metagenomic analysis from manipueira and tucupi

The DNA extraction was performed according to the manufacturer's instructions for the DNA QIAGEN PowerSoil Pro kit (Qiagen, Hilden, Germany). The 16S Metagenomic Sequencing Library Preparation protocol from Illumina (Illumina, San Diego, CA, USA) was used for library construction. The V3 and V4 regions of the 16S ribosomal gene were amplified using polymerase chain reaction (PCR) with universal primers.^30^

The PCR reactions were set up in a final volume of 25 μL, containing 1 μL of DNA (5 ng/μL), 5 μL of buffer 5×, 2 μL of magnesium chloride (MgCl_2_, 25 mm), 1.25 μL of dNTP (2 mm), 0.5 μL of primer S‐D‐Bact‐0341‐b‐S‐17‐N (5′‐CCTACGGGNGGCWGCAG‐3′) (F) (10 mm), 0.5 μL of primer S‐D‐Bact‐0785‐a‐A‐21‐N (5′‐GACTACHVGGGTATCTAATCC‐3′) (R) (10 mm), and 0.15 μL Taq (Promega, Madison, WI, USA). Next, the reactions were amplified in an Applied Biosystems – Veriti 96‐well Thermal Cycler (Foster City, CA, USA) with the following program: initial denaturation at 95 °C for 3 min, 25 cycles of denaturation (95 °C for 30 s), annealing (57 °C for 30 s), extension (72 °C for 30 s) and a final extension (72 °C for 5 min).

A purification step was performed after confirming the amplification of the target amplicons via electrophoresis in 1% agarose gel. The Agencourt AMPure XP magnetic bead kit (Beckman Coulter, Inc., Brea, CA, USA) was used to purify the PCR products according to the earlier‐mentioned 16S library preparation protocol. After the purification step, a second PCR was performed for each sample to add indexes from the Nextera DNA CD Indexes Kit (Illumina). The libraries were once again purified with the Agencourt AMPure XP kit.

For sequencing, the libraries were quantified by fluorimetry using the Qubit dsDNA BR (Broad Range) Assay DNA quantification kit (Thermo‐Fisher Scientific, Waltham, MA, USA) and the Qubit 3.0 fluorimeter (Thermo‐Fisher Scientific). The quality of the libraries was verified by capillary electrophoresis in an Agilent 4200 Tape Station (Agilent Technologies, Santa Clara, CA, USA). The libraries were then standardized to a concentration of 2 nm, following the Illumina protocol recommendations. After normalization, the genomic pool was denatured, and the PhiX sequencing control (20%) was added. The sequencing run was performed on the Illumina MiSeq platform using the MiSeq V3 600‐cycle run kit.

To perform the taxonomic identification, the raw sequences obtained were submitted to the PIMBA (Pipeline for Metabarcoding Analysis) pipeline.^31^ The sequence screening and filtering steps were performed using Prinseq.^32^ Subsequently, the forward and reverse sequences were assembled using the PEAR package.^33^ After assembly, dereplication was performed, amplicon sequence variants (ASVs) were inferred using Swarm 2,^34^ and VSEARCH was used to remove chimeras.^35^ The taxonomy of the ASVs was determined by comparing them with sequences available in the SILVA132 database.^36^ Mitochondrial and chloroplast DNA sequences were removed.

The functional potential analysis was performed, and the ASVs generated were compared to public databases containing information about the genomic, physiological, and ecological functions. The data was processed using Python applications that conduct potential functional annotations for prokaryotes, FAPROTAX – Functional Annotation of Prokaryotic Taxa.^37^

#### Bioactive amines by HPLC

The determination of bioactive amines was carried out by ion‐pair HPLC (LC‐10 AD; Shimadzu, Kyoto, Japan), derivatization with *o*‐phthalaldehyde, and fluorometric detection (340 and 445 nm of excitation and emission, respectively), according to Brito *et al*.^5^ A Luna column (C18, 4.6 mm × 250 mm, 5 μm; Phenomenex, Torrance, CA, USA) and a pre‐column (C18, 4 mm × 3 mm) were used. A gradient of 0.2 m sodium acetate and 0.3 mm sodium octane sulfonate at pH 4.9 (mobile phase 1) and acetonitrile (mobile phase 2) was used. Ten amines were identified by comparison of retention times and co‐elution with standards. The quantification was performed by interpolation in external analytical curves.

#### Statistical analysis

All analyses were performed in triplicate using a completely randomized design, and the results are presented as mean ± standard deviation. The results from the physicochemical analyses and biogenic amines were evaluated through analysis of variance (ANOVA), and the means were compared by the Tukey test (*P* < 0.05). Multivariate analyses (principal component analysis (PCA) and hierarchical cluster analysis (HCA)) were performed, using the physicochemical results and biogenic amines as active variables. Hierarchical clusters were obtained using Ward's method. Statistical analysis was performed using the Past 4.03 software (freeware license).^38^

Metagenomic analyses of the samples were performed to assess alpha diversity (Shannon index), richness (Chao1), and community structure (Simpson index). The Kruskal–Wallis test was applied with a significance threshold of *P* < 0.05 using RStudio 4.0 software (2020). Graphs for alpha diversity, richness, structure, and functional potential were generated using the *Phyloseq* and *ggplot2* packages,^39^, ^40^ as well as Reshape2 and Dplyr packages.^40^, ^41^, ^42^

## RESULTS

### Physicochemical characteristics during the manipueira fermentation

To ensure that the fermentation of manipueira, both the spontaneous (CT) and the treatment with *P. acidilactici* as starter culture (Pa), followed their due course, and the physicochemical characteristics were determined (Table 1). The main changes (Tukey test, *P* ≤ 0.05) included decreases in pH, total and reducing sugars, and total solids. In addition, there was an increase in total titratable acidity, whereas soluble solids increased up to 12 h, decreasing afterward.

**Table 1:** Changes in pH, total titratable acidity (TTA), reducing sugars (RS), total sugars (TS), and soluble solids (SS) during manipueira fermentation without starter culture (control treatment, CT) and with *Pediococcus acidilactici* (Pa) starter culture inoculation and of the respective tucupi

Manipueira fermentation time (h)	pH		TTA (meq LA 100 mL^−1^)^†^		RS (g L^−1^)		TS (g L^−1^)		SS (°Brix)	
	CT	Pa	CT	Pa	CT	Pa	CT	Pa	CT	Pa
0	4.40 ± 0.17^aA^	4.44 ± 0.20^aA^	0.19 ± 0.04^fA^	0.18 ± 0.03^eA^	2.08 ± 0.01^aB^	2.83 ± 0.03^aA^	4.13 ± 0.07^aA^	4.03 ± 0.16^aB^	6.00 ± 0.01^dA^	6.00 ± 0.01^cdA^
4	3.74 ± 0.01^bA^	3.71 ± 0.01^bA^	0.31 ± 0.01^eA^	0.29 ± 0.01^dA^	2.05 ± 0.01^bA^	1.33 ± 0.01^bB^	3.69 ± 0.21^bA^	3.57 ± 0.04^bB^	5.80 ± 0.01^dA^	6.00 ± 0.35^cdA^
8	3.73 ± 0.01^bA^	3.68 ± 0.01^bB^	0.37 ± 0.02^deA^	0.34 ± 0.01^dB^	1.15 ± 0.01^dA^	0.66 ± 0.01^eB^	3.17 ± 0.04^cA^	2.52 ± 0.02^cB^	5.80 ± 0.01^dA^	5.50 ± 0.01^eA^
12	3.58 ± 0.01^bcA^	3.56 ± 0.01^bcA^	0.43 ± 0.01^cdB^	0.44 ± 0.01^cA^	1.29 ± 0.01^cA^	1.24 ± 0.01^cB^	2.35 ± 0.03^dA^	1.96 ± 0.03^dB^	7.90 ± 0.01^aA^	6.92 ± 0.01^abA^
16	3.49 ± 0.01^cdA^	3.45 ± 0.01^cdA^	0.51 ± 0.05^bcB^	0.55 ± 0.03^bA^	0.81 ± 0.01^eA^	0.54 ± 0.01^fB^	2.08 ± 0.01^eA^	1.90 ± 0.01^dB^	6.70 ± 0.12^cA^	5.67 ± 0.06^deB^
20	3.35 ± 0.01^deB^	3.45 ± 0.01^cdA^	0.33 ± 0.03^deB^	0.36 ± 0.01^dA^	0.45 ± 0.01^gA^	0.37 ± 0.01^gB^	1.04 ± 0.01^gA^	0.84 ± 0.01^eB^	5.30 ± 0.01^eA^	6.60 ± 0.01^bB^
24	3.34 ± 0.02^deA^	3.29 ± 0.01^dB^	0.57 ± 0.03^bA^	0.51 ± 0.01^bcB^	0.57 ± 0.01^fA^	0.54 ± 0.01^fB^	1.21 ± 0.01^fA^	0.83 ± 0.01^eB^	6.00 ± 0.17^dA^	6.20 ± 0.01^cA^
Tucupi (after boiling)	3.26 ± 0.01^eA^	3.28 ± 0.01^dA^	0.89 ± 0.01^aB^	1.06 ± 0.51^aA^	1.15 ± 0.10^dA^	1.12 ± 0.01^dA^	2.27 ± 0.01^dA^	2.11 ± 0.03^dB^	7.20 ± 0.01^bA^	7.00 ± 0.02^aB^

^†^ Milliequivalent lactic acid per 100 milliliters of sample.

Mean ± standard deviation with different lowercase letters in the same columns (fermentation time) and different capital letters in the same line (treatments – CT and Pa) are significantly different (Tukey test, *P* ≤ 0.05).

Significant changes were observed between the fermentation treatments (CT and Pa) regarding some physicochemical parameters. The pH was higher for CT at 8 and 24 h fermentation (*P* ≤ 0.05) compared to Pa. Whereas, total titratable acidity was lower for CT compared to Pa, at 8, 12, 20 and 24 h fermentation. Reducing and total sugars were higher in CT compared to Pa. Total solids were higher for CT compared to Pa at 16 and 20 h. Based on these results, adding starter culture prior to manipueira fermentation led to greater and faster consumption of fermentable sugars.

### Metagenomic analysis

**Figure 1:**
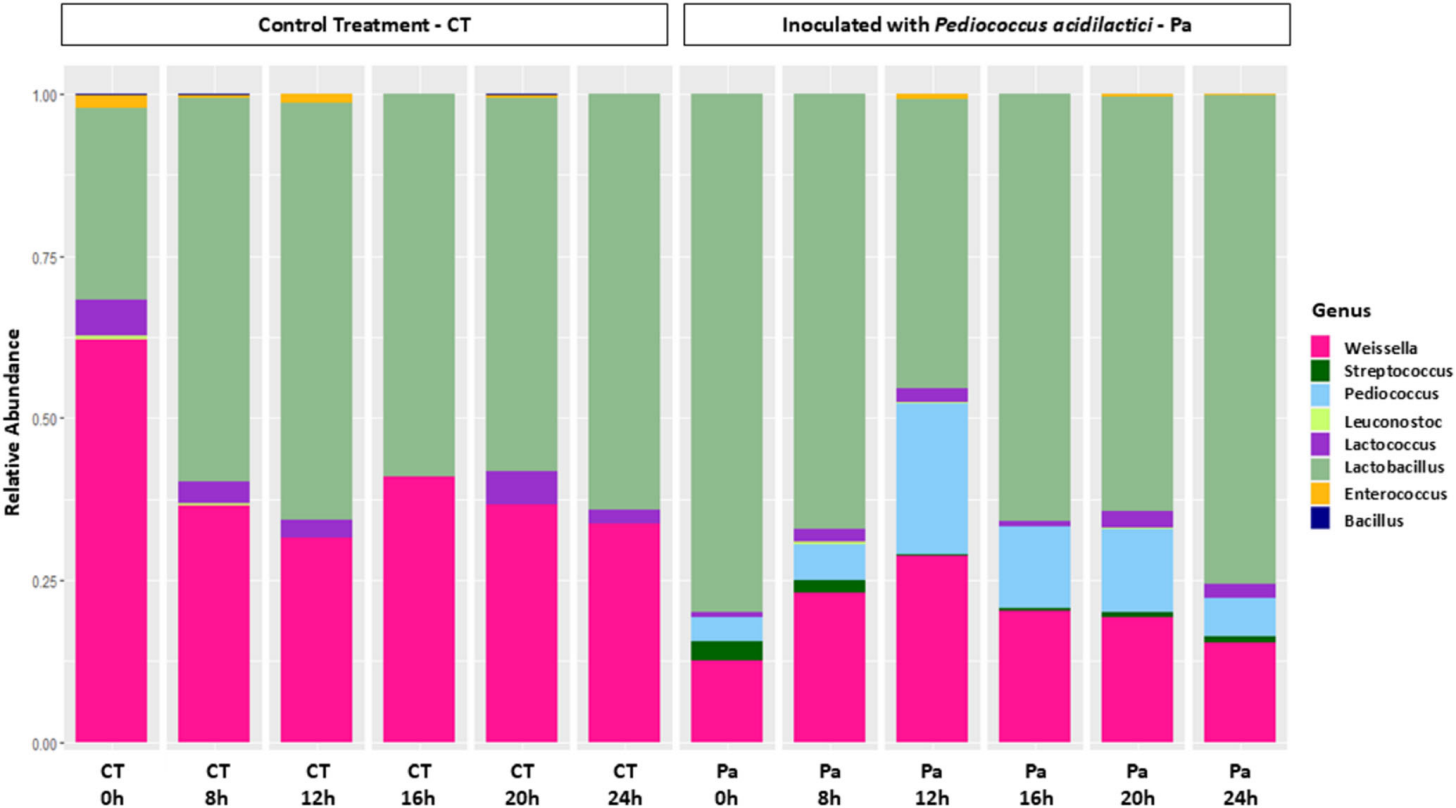
Relative abundance of microbial genera during manipueira fermentation without starter culture (control treatment, CT) and with the inoculation of *Pediococcus acidilactici* (Pa) as starter culture. The graph shows the shifts in microbial composition across fermentation time points, highlighting the dominance of lactic acid bacteria in the inoculated treatment. These changes illustrate how starter addition influences microbial succession, promoting the prevalence of beneficial fermentative taxa and accelerating the stabilization of the microbial community.

The microbial diversity found in the fermented cassava is indicated in Fig. 1. Overall, eight bacterial genera were identified, including *Weissella*, *Streptococcus*, *Pediococcus*, *Leuconostoc*, *Lactococcus*, *Lactobacillus*, *Enterococcus*, and *Bacillus*, showing the predominance of the Firmicutes phylum.

In the CT, throughout fermentation, six genera were observed, for example *Weissella*, *Lactobacillus*, *Lactococcus*, *Enterococcus*, *Leuconostoc*, and *Bacillus*. The fresh manipueira (time 0) had a prevalence of *Weissella* (~60%), followed by *Lactobacillus* (~29%), and *Lactococcus* (~6%). Whereas *Enterococcus*, *Bacillus*, and *Leuconostoc* had a low relative distribution (≤ 2%). The microbial composition changed throughout the fermentation, showing microbial dynamics typical of a fermentation process. There was a decrease in the abundance of *Weissella* over time, reaching a lower occurrence at 12 h and remaining constant until 24 h (~30%). On the contrary, *Lactobacillus* occurrence increased to 60% prevalence from 8 h on. The occurrence of *Lactococcus*, *Enterococcus*, and *Leuconostoc* was sporadic and variable, keeping percentages below 10%. At the end of fermentation, in the CT, there was a predominance of *Lactobacillus* (64%), followed by *Weissella* (34%) with traces of *Lactococcus*.

When fermentation was undertaken in the presence of the starter culture (Pa treatment), the same genera were observed, except for *Bacillus*, which was not found. However, Pediococcus and Streptococcus were also found to be different from the CT fermentation. There was also a greater relative abundance of *Lactobacillus* (~70%), which decreased, reaching a minimum at 12 h, and increased again to initial values. On the contrary, the prevalence of *Weissella* and *Pediococcus* (Fig. 1) increased to 12 h, decreasing afterward to initial values. During fermentation, the occurrences of *Lactococcus*, *Streptococcus*, and *Enterococcus* were observed in a random way, at percentages below 3%. At the end of fermentation, there was a predominance of *Lactobacillus* (75%), followed by *Weissella* (15%) and *Pediococcus* (6%).

### Bioactive amines during the fermentation of manipueira and in tucupi

Only putrescine and histamine, out of the ten amines analyzed, were detected in manipueira and tucupi from both treatments (Table 2). During CT fermentation, putrescine levels varied from 1.72 to 2.60 mg kg^−1^, with no significant difference (*P* > 0.05) observed throughout fermentation or in tucupi. In the Pa treatment, putrescine levels varied from 1.68 to 2.77 mg kg^−1^, with a significant difference observed only at 24 h of fermentation compared to initial levels.

**Table 2:** Levels of free bioactive amines^†^ (mg kg^−1^) during manipueira fermentation without starter culture (control treatment, CT) and with *Pediococcus acidilactici* starter culture (Pa) and the respective tucupi

Treatment	Manipueira fermentation time (h)							Tucupi
	0	4	8	12	16	20	24	(after boiling)
Putrescine								
CT	2.16 ± 0.47^abA^	1.72 ± 0.02^bA^	2.06 ± 0.00^abA^	2.15 ± 0.14^abA^	2.43 ± 0.25^abA^	2.43 ± 0.02^abA^	2.60 ± 0.02^aA^	2.03 ± 0.05^abA^
Pa	1.81 ± 0.08^bcA^	1.68 ± 0.07^cA^	1.88 ± 0.10^bcA^	1.90 ± 0.18^bcA^	2.32 ± 0.20^abcA^	2.45 ± 0.07^abA^	2.77 ± 0.18^aA^	2.66 ± 0.30^aA^
Histamine								
CT	0.45 ± 0.01^dA^	0.53 ± 0.11^dA^	0.59 ± 0.01^dB^	0.87 ± 0.13^cdB^	1.34 ± 0.31^bcB^	1.66 ± 0.16^abB^	2.13 ± 0.17^aB^	1.57 ± 0.05^abB^
Pa	0.57 ± 0.16^dA^	0.59 ± 0.01^dA^	1.25 ± 0.06^cdA^	2.05 ± 0.30^cA^	3.92 ± 0.41^bA^	5.17 ± 0.09^aA^	5.78 ± 0.25^aA^	5.06 ± 0.49^aA^

^†^ Only two out of nine amines were detected. Tyramine, histamine, serotonin, cadaverine, agmatine, spermidine, and tryptamine were not detected in any sample (limit of quantification (LOQ) = 0.04 mg kg^−1^).

Mean value ± standard deviation for each amine with different lowercase letters in the same line (fermentation time) and different capital letters in the same columns (treatments) for each amine are significantly different (Tukey test, *P* ≤ 0.05).

Histamine levels varied from 0.45 to 2.13 mg kg^−1^ in CT, and from 0.57 to 5.78 mg kg^−1^ in Pa. Significant differences (*P* ≤ 0.05) in histamine levels were observed during fermentation between treatments. The increases in histamine became significant at 16 h for CT and 12 h for Pa. Thus, Pa treatment led to earlier histamine synthesis and higher histamine accumulation compared to CT. Differences in histamine levels between treatments were detected from 8 h of fermentation onward, with significantly higher levels in Pa than in CT.

In tucupi, putrescine levels did not differ significantly between treatments, but histamine levels were higher in Pa compared to CT.

### Differentiation of manipueira fermentation processes using multivariate analysis

**Figure 2:**
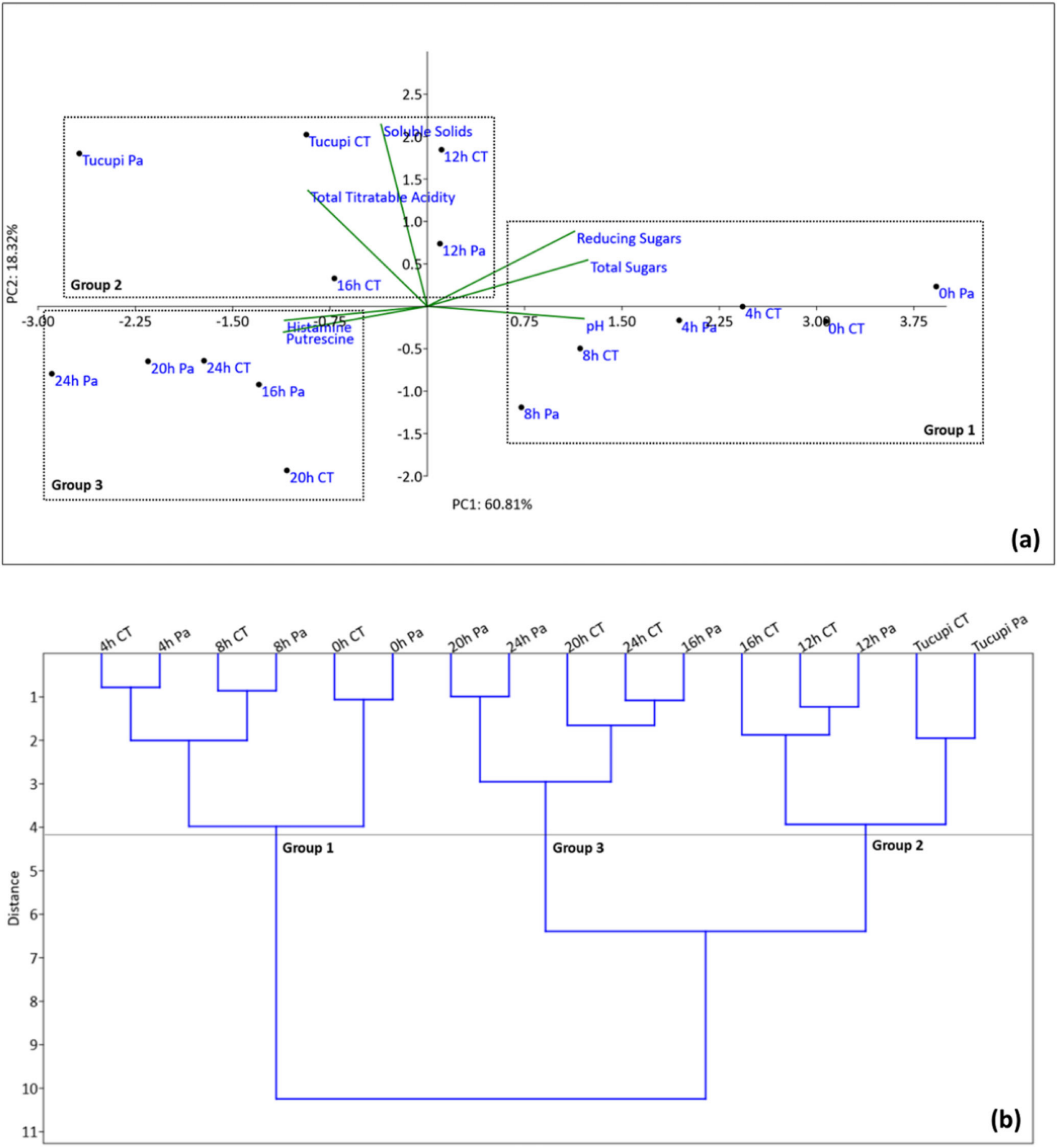
Principal component analysis (a) and hierarchical cluster analysis (b) of the physicochemical characteristics of manipueira during fermentation without starter culture (control treatment, CT) and with the inoculation of *Pediococcus acidilactici* (Pa) as a starter culture, as well as the respective final product – tucupi.

To differentiate the fermentation processes of manipueira (CT and Pa), PCA and HCA were performed (Fig. 2). Two components accounted for approximately 79.13% of the variation (PC1 + PC2). In both treatments, hierarchical group formation (HCA) was observed, allowing the differentiation of three groups based on the active variables (physicochemical characteristics and amines). Group 1 clustered manipueira from 0 to 8 h fermentation of both treatments, which represents the initial fermentation stage, characterized by higher pH and sugars (total and reducing), and lower levels of biogenic amines. In group 2, intermediate fermentation samples (12 and 16 h) were clustered along with the tucupi from both treatments. Pa treatment at 16 h did not fit into this group, suggesting that the use of *P. acidilactici* accelerated fermentation. This group was characterized by higher soluble solids and total titratable acidity. In Group 3, samples from the fermentation times of 20 and 24 h for both treatments and CT at 16 h fermentation. This group showed higher putrescine and histamine.

## DISCUSSION

### Physicochemical characteristics during the manipueira fermentation

These changes are typical of manipueira fermentation for tucupi production.^3^, ^5^ The sugar contents decrease, possibly due to its metabolism by fermentation microorganisms, such as LAB,^5^, ^43^ and due to the hydrolysis of non‐reducing sugars.^5^, ^17^, ^44^ Organic acids are produced,^6^ causing a decrease in pH and an increase in the acidity of the medium, which is important as it contributes to the preservation of the fermented product.^45^ The increase in soluble solids observed in both treatments (Pa and CT) during manipueira fermentation was also reported by Brito *et al*.,^5^ who attributed it to organic acids released during fermentation. These acids lower the pH and increase the total titratable acidity. Organic acids are water‐soluble substances derived from carbohydrate metabolism by fermentative microorganisms.^43^

The physicochemical characteristics of tucupi (Table 1) were similar for both treatments, except for total titratable acidity and total sugars, which were higher and lower, respectively (Tukey test, *P* ≤ 0.05) when the starter culture (Pa) was used. The pH values complied with legislation, which ranges from 3.5 to 4.3.^7^ Nevertheless, the acidity in the Pa treatment exceeded the maximum value recommended (0.8 meq lactic acid (LA) 100 mL^−1^). Since manipueira at 24 h had total titratable acidity ≤ 0.57 meq LA 100 mL^−1^, it is possible that the heat treatment was responsible for the increased values, not the fermentation step. A significant difference (Tukey test, *P* < 0.05) was observed between the treatments regarding total sugar contents, with lower levels observed in the treatment with added inoculum (Pa). However, both are within the limit (< 15 g L^−1^) recommended by the Brazilian legislation.^7^

### Metagenomic analysis

Among the predominant genera, Lactobacilli are Gram‐positive bacteria with negative reactions to catalase, non‐motile, and non‐spore‐forming rods or coccobacilli. It can be homo‐ or hetero‐fermentative, converting carbohydrates into lactic acid, acetic acid, ethanol, and carbon dioxide (CO_2_). Lactobacilli are associated with food and health; they have probiotic potential, inhibit pathogens, modify the gut microbiota, and modulate the host immune system. *Lactobacillus* has a long history of safe use being generally recognized as safe (GRAS).^46^

*Weissella* (Gram‐positive, catalase‐negative, non‐spore‐forming, coccoid morphology, or short bacilli) is a LAB. They have been isolated from fermented foods, such as cheeses, fermented vegetables, and fermented milk. Some strains can produce exopolysaccharides and non‐digestible oligosaccharides. *Weissella* can produce bacteriocins and hydrogen peroxide. They have great potential for use in the food industry.^47^

The genus of the starter culture used, *Pediococcus*, has been widely used in the production of traditional fermented foods enabling distinct characteristics. They can produce bacteriocins and pediocins, they are potential bio‐preservatives with a large inhibitory spectrum and they show probiotic properties.^22^

However, some of the described bacterial genera can have potentially pathogenic species, including *Bacillus*, *Enterococcus*, and *Streptococcus* (Fig. 1). Some *Bacillus* can be pathogenic, for example, *Bacillus cereus*, which can contaminate starchy foods.^48^ In addition, some *Bacillus* species can produce toxins, such as Enterotoxin T (BceT) and Enterotoxin FM (EntFM), which can induce acute diarrheal episodes, disruption of the gut microbiota, and emesis when present at high concentrations.^49^ However, others can degrade cyanogenic compounds, which are present in some cassava species and can be toxic to humans.^26^, ^50^

The *Enterococcus* and *Streptococcus* genera are commonly associated with fecal contamination and, therefore, indicative of poor hygienic‐sanitary conditions.^14^ Cavalcante *et al*.^26^ detected the presence of *Streptococcus* during manipueira fermentation, suggesting as a possible source the origin of the raw material, which is in direct contact with the soil and typically does not undergo a sanitization process to eliminate the native microbiota. In addition, fresh cassava is commercialized in open‐air markets, which may also be subject to cross‐contamination.^5^ These possible sources of contamination cannot be overlooked. *Enterococcus faecalis* is frequently detected in cassava, and its presence is considered a potential health risk, as high concentrations are associated with the transmission of opportunistic infections to humans.^51^

The use of *P. acidilactici* as a starter culture represents a promising strategy for application in tucupi production, offering a potential approach to ensure food safety by inhibiting the growth of microorganisms responsible for foodborne illnesses, as observed for *Bacillus*, which was not detected in the Pa treatment, and for the lower occurrence of *Enterococcus*. In addition, in the Pa treatment, there was an increase in the prevalence of *Lactobacillus*. This microbial proliferation was accompanied by the accumulation of histamine, suggesting the occurrence of complex microbial interactions with implications for biogenic amine production. The growth of *Lactobacillus* can be partially attributed to the rapid acidification of the medium with the addition of *P. acidilactici*, which established an environment conducive to the survival and proliferation of acid‐tolerant LAB.^15^, ^52^ The observed pH reduction, in association with the potential release of free‐amino acids – particularly histidine – via direct or indirect proteolytic activity of the starter culture, may have activated metabolic pathways involved in the biosynthesis of this biogenic amine by LAB.^53^ Species of *Lactobacillus*, including *Lactobacillus buchneri*, *Lactobacillus parabuchneri*, and *Lactobacillus hilgardii*, have been identified as potential histamine producers, especially under fermentative conditions where histidine is available, and the pH is acidic.^54^ The expression of the enzyme histidine decarboxylase (HDC), which catalyzes the conversion of histidine into histamine, is known to be enhanced under such environmental conditions.^55^ Furthermore, the concurrent growth of *Weissella*, a genus comprising species with histamine‐producing potential such as *Weissella cibaria* and *Weissella confusa*, supports the hypothesis of a synergistic microbial contribution to histamine accumulation.^52^

Metagenomic analysis provides time‐resolved insight into the microbial succession that drives tucupi fermentation, capturing the shift from early, acid‐sensitive communities to LAB – dominated consortia. For the first time, methods of metagenomic analysis were performed to conduct a detailed investigation of the microbial diversity in manipueira fermentation, revealing key aspects such as the predominance of specific or more genera, microbial succession, and interactions. Additionally, these methods help identify potential pathogens and spoilage agents, facilitating the development of strategies to enhance food production and ensure quality and safety. They also aid in identifying biomarkers that contribute to food quality assessment and the formulation of optimal starter cultures.^56^

**Figure 3:**
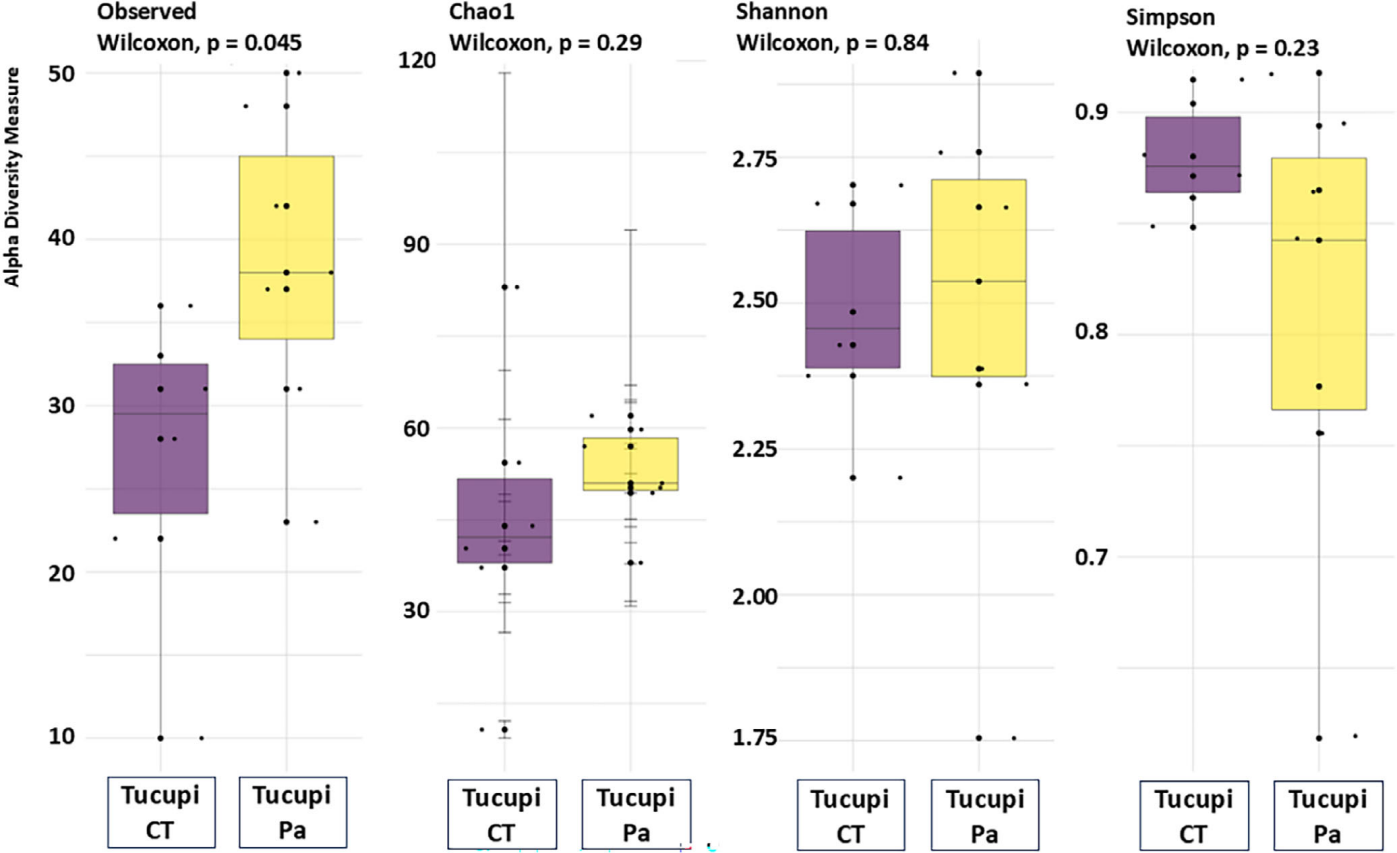
Richness estimator (Chao1) and diversity indices (Shannon index and Simpson index) of microbial genera during manipueira fermentation without starter culture (control treatment, CT) and with the inoculation of *Pediococcus acidilactici* (Pa) as starter culture. The Chao1 estimator indicates differences in species richness between treatments, while the Shannon and Simpson indices reflect shifts in microbial diversity and evenness throughout fermentation. The patterns demonstrate that inoculation leads to reduced overall diversity but promotes dominance of beneficial taxa, contributing to a more controlled and predictable fermentation process.

**Figure 4:**
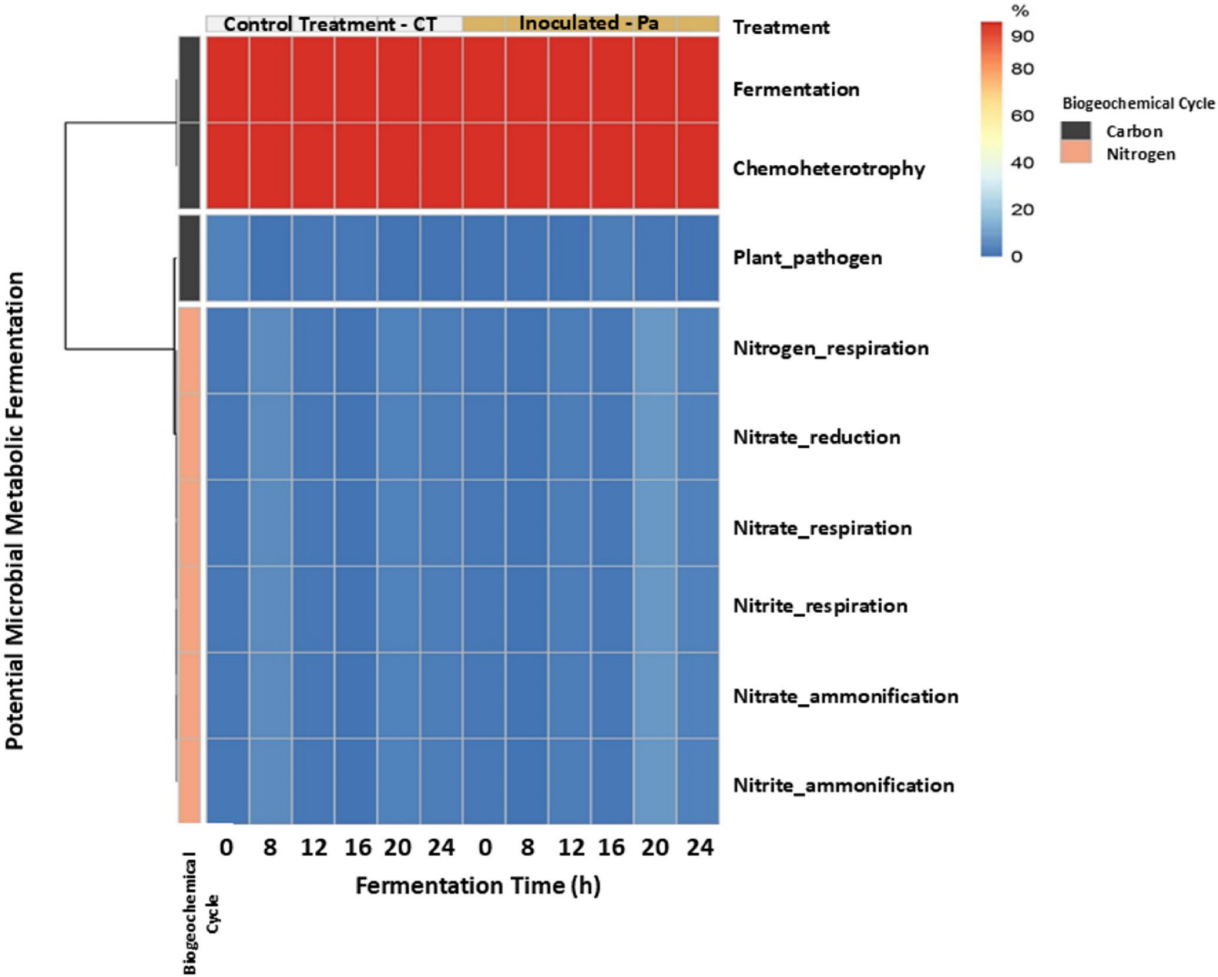
Potential microbial metabolic functions (fermentation and biogeochemical cycle) of the microbial genera identified during manipueira fermentation without starter culture (control treatment, CT) and with the inoculation of *Pediococcus acidilactici* (Pa) as starter culture.

Despite variation in microbial diversity during fermentation and across treatments, these differences were not statistically significant, as demonstrated by alpha‐diversity analysis using Chao1 (taxon richness), Shannon index (species diversity), and Simpson index (community structure) estimators (Fig. 3). The identified genera were associated with chemoheterotrophic, fermentation, and nitrogen‐related respiration processes (Fig. 4). Based on taxonomic identification and database information, the LAB involved in manipueira fermentation were predominantly chemoheterotrophic (97.2% of the identified population). These microorganisms derive energy by utilizing electrons from hydrogen atoms of organic compounds. Members of the genera *Lactobacillus* and *Bacillus*, for example, participate in both homofermentative and heterofermentative pathways. In the homofermentative pathway, they produce only lactic acid; in the heterofermentative pathway, they generate additional organic compounds, such as diacetyl, acetic acid, and ethanol, among others.^57^ However, the genera *Leuconostoc* and *Weissella*, for example, are obligatory heterofermentative and produce lactic acid, CO_2_, ethanol, and/or acetate via the hexose‐monophosphate and phosphoketolase pathways.^58^ This fact can directly influence the physicochemical and sensory characteristics of the product. Finally, the genera *Enterococcus*, *Lactococcus*, *Streptococcus*, and *Pediococcus* are obligatory homofermentative and produce lactic acid.^59^

Additionally, the *Lactobacillus* genus appears to be involved in nitrogen‐related respiration processes, including nitrite and nitrate ammonification and nitrite respiration (Fig. 4). According to Zotta *et al*.,^60^ certain *Lactobacillus* species, particularly *Lactobacillus plantarum*, have been reported in manipueira fermentation by Brito *et al*.,^61^ are capable of aerobic respiration. Tejedor‐Sanz *et al*.^62^ observed that *Lactobacillus plantarum* can consume electrons from a cathode and couple the oxidation reaction to the reduction of both an endogenous organic electron acceptor (pyruvate) and an exogenous inorganic electron acceptor (nitrate). This process redirects glucose fermentation toward lactate degradation, enhancing cell viability even when there is sugar exhaustion. Such metabolic flexibility may explain the predominance of the *Lactobacillus* genus in manipueira fermentation and other cassava‐based fermented products, even when fermentable carbohydrates are scarce.

### Bioactive amines during the fermentation of manipueira and in tucupi

Previous studies^5^ also reported the presence of putrescine in fresh manipueira, which could be naturally present in cassava or result from inadequate hygienic‐sanitary conditions during processing.^14^ However, histamine was not detected by Brito *et al*.^5^ until the 16th day of manipueira fermentation, suggesting its production during this process.

Additionally, the results indicated that the heat treatment (cooking) did not affect the putrescine and histamine levels in fermented manipueira. The thermal stability of histamine has been previously reported, supporting our findings.^5^, ^10^

The production of biogenic amines in fermented foods can occur due to the presence of microorganisms with amino acid decarboxylase activity.^53^ The first significant increase in histamine during fermentation took place at pH 3.5 and kept increasing. It is possible that the low pH (~3.5) triggers free amino acids decarboxylase activity forming amines (e.g., histidine and histamine, respectively) to help buffer the media.^23^, ^53^ In this way, the production of amines may serve as a protective mechanism against the acidic environment, a characteristic of fermentation, which can be harmful to microorganisms.^10^ It could also be a response to nutritional stress.^23^

The physicochemical findings for pH in this study (from 4.40 to 3.34 in CT and from 4.44 to 3.29 in the Pa treatment), together with the microbial genera identified by metagenomic analysis, indicate that, during manipueira fermentation, the conditions were favorable for the activity of HDC for bacteria belonging to the genera *Lactobacllus*, *Pediococcus*, and *Streptococcus*.^10^ Cavalcante *et al*.^26^ further reported that the availability of free amino acids released during proteolysis throughout manipueira fermentation may also contribute to the formation of bioactive amines.

The genera relative abundances (Fig. 1) can explain the higher histamine values in the Pa treatment (Table 2). Some LAB genera, including *Lactobacillus* (*Lactobacillus plantarum* and *Lactobacillus hilgardii*), are associated with putrescine production.^11^, ^14^ However, the literature also supports the effectiveness of *P. acidilactici* strains in reducing putrescine levels in fermented products,^19^ which was not the case in this study. Conversely, histamine levels were higher in the inoculated treatment (Pa).

The presence of certain biogenic amines, particularly histamine, in foods can represent a health concern. At high levels, histamine can lead to histamine intoxication.^13^, ^14^ However, even at very low levels, histamine can cause histamine intolerance, which is characterized by an impaired ability to metabolize histamine, such as reduced synthesis of diamine oxidase.^13^ Therefore, it would be interesting to investigate the ability of the starter culture to encode the HDC gene as a screening tool.^63^ In addition, some starters can degrade histamine,^64^, ^65^ which could warrant low histamine levels in the product.^66^

In our study, putrescine was consistently detected at low concentrations (2.01–2.66 mg kg^−1^), remaining constant throughout fermentation. This stability suggests that *P. acidilactici* may have contributed to no statistical difference of growth of putrescine producing microorganisms, since previous studies on manipueira fermentation reported higher and increasing putrescine levels over time.^5^ In contrast, histamine showed a gradual increase in the Pa treatment, coinciding with the predominance of acid‐tolerant genera such as *Lactobacillus* and *Weissella*. Both genera are known to include strains harboring the HDC gene, which catalyzes the conversion of histidine into histamine.^5^, ^10^, ^14^, ^67^ These observations suggest that the microbial shifts driven by the addition of a starter culture modulated the pattern of histamine accumulation. However, the maximum histamine levels detected in this study (~5.8 mg kg^−1^) were well below the recommended limits of 50 mg kg^−1^ for histamine intoxication.^14^ Nevertheless, it would be a problem for individuals with histamine intolerance.

In the metagenomic analysis of our study, the genera *Weissella* and *Lactobacillus* were active throughout the entire fermentation period. These genera can produce histamine in significant quantities; however, modification in the HDC gene can reduce its production.^68^ This underscores the importance of selecting starter cultures that lack histamine‐producing capacity. In this context, the use of *P. acidilactici* as a starter culture remains promising, provided that future studies in the Amazon focus on screening isolates without HDC activity. Additionally, mitigation strategies could include the use of co‐cultures combining *P. acidilactici* with strains known to degrade histamine. Overall, our findings reinforce the need for further optimization of starter culture selection to ensure both safety and quality in tucupi production.

As a traditional food of the Amazon region, tucupi is produced through artisanal practices that often lack standardization in aspects such as good manufacturing practices, hygiene, cooking time for elimination of the natural HCN of cassava roots, and storage conditions.^3^, ^6^ Many small‐scale producers follow their own protocols to preserve product identity, which generally excludes preliminary sanitization of cassava roots. This omission may be explained by the potential impact of sanitization on the naturally occurring microbial population, as it could reduce or eliminate fermentative microorganisms essential for manipueira and other cassava‐derived products, such as fermented cassava flour.

Tucupi production still lacks standardized hygienic‐sanitary practices, with manual handling and inconsistent utensil cleaning potentially leading to microbial carryover and spontaneous fermentation. Brito *et al*.^5^ detected only four amines – spermidine, tyramine, histamine, and putrescine – in laboratory‐scale manipueira fermentations, with putrescine showing progressive accumulation (3.88–5.40 mg kg^−1^). In contrast, putrescine levels in this study remained low (2.01–2.66 mg kg^−1^), suggesting adequate raw material conditions and effective control by *P. acidilactici* starter culture. The analytical limit of quantification (LOQ, 0.04 mg kg^−1^) confirmed very low or undetectable levels of other amines.

Governments should establish action plans and preventive hygienic‐sanitary measures to reduce microbial populations that favor the formation of undesirable compounds, such as biogenic amines (e.g., putrescine, cadaverine, and histamine). In addition, appropriate storage conditions are required to ensure microbiological and physicochemical stability, as well as to provide a better understanding of amine accumulation during tucupi shelf life and commercialization. Currently, tucupi is predominantly marketed at fairs and sold at ambient temperature, as a historical practice that underscores the need to raise consumer awareness and demand for safe, high‐quality products through targeted governmental initiatives. Therefore, the present findings are highly relevant in an industrial context.

### Differentiation of manipueira fermentation processes using multivariate analysis

A negative correlation was observed between pH and the two biogenic amines identified (putrescine and histamine), showing an inversely proportional relationship (*r* = −0.55 and *r* = −0.58, respectively). This is due to the microorganisms present in the fermentation medium, which decarboxylate the amino acids from the raw material as a mechanism to remain active and viable in the medium. As a result, the acid production, typical of these products, favors this reaction.^10^, ^14^, ^69^

The application of multivariate analysis tools (PCA and HCA) was chosen to better understand the complexity of microbial community dynamics and physicochemical parameters throughout tucupi fermentation. PCA allowed the visualization of correlations between samples and variables, revealing how specific microbial genera and chemical markers contributed to the differentiation of the fermentation stages. HCA complemented this approach by grouping samples based on similarity, confirming the trends observed in the PCA plots.

Potential active variables, such as pH variation, fermentation time, and the heterogeneous nature of manipueira as a raw material, were considered when interpreting these results. These variables can influence both microbial succession and metabolite production, possibly affecting clustering patterns. Nonetheless, the consistent grouping of replicates within treatments supports the robustness of the multivariate analyses. Therefore, PCA and HCA provided complementary and reliable insights into how starter inoculation modulated microbial diversity and biochemical changes during tucupi fermentation.

These results represent the first study performing metagenomic analysis to investigate the microbial dynamics during tucupi production and the influence of an Amazonian LAB starter. These results are promising, as the use of starter cultures can modulate fermentation time and the accumulation of biogenic amines. This approach offers potential benefits for the food industry by improving process standardization, product safety, and quality control in tucupi production.

## CONCLUSIONS

Manipueira fermentation, both spontaneous and with *P. acidilactici* as a starter, followed expected patterns of a decrease in pH and sugars and an increase in total titratable acidity. The microbial dynamics during spontaneous manipueira fermentation, determined by metagenomic analysis, indicated the prevalence of *Weissella* in fresh manipueira, followed by *Lactobacillus*. However, during fermentation, *Lactobacillus* was prevalent, followed by *Weissella. Lactococcus*, *Enterococcus*, and *Bacillus* were also present at lower incidence. When *P. acidilactici* was used, there was a higher prevalence of *Lactobacillus* and a lower prevalence of *Weissella. Pediococcus* occurrence increased up to 12 h, decreasing afterward. *Bacillus* was not found, whereas *Streptococcus* was present as a minor constituent. The use of *P. acidilactici* accelerated the fermentation and modulated the microbiota, decreasing the prevalence of potentially pathogenic bacteria (e.g., *Bacillus*).

Among the ten bioactive amines investigated, only putrescine and histamine were detected. No significant difference was observed for putrescine during fermentation and between treatments. However, histamine levels increased with fermentation time, and higher levels were found when *P. acidilactici* was used compared to the control. Results suggest that the increased prevalence of *Lactobacillus* could be responsible for the higher histamine levels, which deserve further studies.

Multivariate analysis (PCA and HCA) confirmed the results and differentiated samples into three clusters: initial, intermediate, and final fermentation, in which *P. acidilactici* accelerated the fermentation. However, studies are needed to prevent histamine formation and build‐up.

This study provides an overview of the benefits of using a starter culture in tucupi production and its influence on fermentation time and modulation of the microbiota. Further studies are needed to obtain an ideal inoculum (other species of LAB) or even different microorganisms to optimize fermentation and minimize the build‐up of undesirable amines. These efforts will enhance the cassava‐based product supply chain, thus strengthening both local science and the economy. These future studies may support the development and adoption of public policies aimed at raising awareness and enhancing the value of tucupi and its production chain.

## CONFLICT OF INTEREST

The authors declare that they have no conflicts of interest.

## Data Availability

The data that support the findings of this study are available from the corresponding author upon reasonable request.
